# Identification of a novel prognostic signature for HCC and analysis of costimulatory molecule-related lncRNA AC099850.3

**DOI:** 10.1038/s41598-022-13792-z

**Published:** 2022-06-15

**Authors:** Qi Wang, Qiong Fang, Yanping Huang, Jin Zhou, Meimei Liu

**Affiliations:** Department of Histology and Embryology, Anhui Medical College, Hefei, 230601 Anhui China

**Keywords:** Cancer, Cell biology, Computational biology and bioinformatics, Molecular biology

## Abstract

Costimulatory molecules are involved in initiation of anti-tumor immune responses while long non‐coding RNAs (lncRNAs) regulate the development of various cancers. However, the roles of lncRNA in hepatocellular carcinoma (HCC) have not been fully established. In this study, we aimed at identifying lncRNAs-related costimulatory molecules in HCC and to construct a prognostic signature for predicting the clinical outcomes for HCC patients. Data were downloaded from The Cancer Genome Atlas database for bioinformatics analyses. Costimulatory molecules were obtained from published literature. The R software, SPSS, and GraphPad Prism were used for statistical analyses. A risk model that is based on five costimulatory molecule-related lncRNAs was constructed using lasso and Cox regression analyses. Multivariate regression analysis revealed that the risk score could predict the prognostic outcomes for HCC. Samples in high- and low-risk groups exhibited significant differences in gene set enrichment and immune infiltration levels. Through colony formation and CCK8 assays, we found that AC099850.3 was strongly associated with HCC cell proliferation. We identified and validated a novel costimulatory molecule-related survival model. In addition, AC099850.3 was found to be closely associated with clinical stages and proliferation of HCC cells, making it a potential target for HCC treatment.

## Introduction

Liver cancer is one of the deadliest cancers and the third most prevalent tumors, with about 905,000 new diagnosed cases and 830,000 deaths worldwide in 2020^1^. Hepatocellular carcinoma (HCC), the most common primary liver cancer (PLC) subtype, accounts for over 90% of all liver cancer cases. Currently, there is no targeted therapy for HCC, and the main treatment options include immunotherapy and multi-tyrosine-kinase inhibitors^2–4^. Although many advances have been made in diagnostic modalities and standard treatments for HCC, recurrence and death rates are still high, with an estimated 5-year overall survival rate of about 12% during the past two decades^5,6^. Therapeutic outcomes for HCC patients, especially early-stage liver cancer patients, largely rely on the time interval from diagnosis to the time of curative treatment initiation (TTI), thus, early diagnosis and initiation of treatment is of great importance^7^. However, most HCC patients are diagnosed in intermediate and advanced stages, thereby depriving them the best treatment opportunities, resulting in poor prognostic outcomes^8,9^. Therefore, there is a need to identify effective prognostic markers, with the overarching goal of improving clinical outcomes.

Immunotherapy, an additional treatment option for cancer patients, is a promising therapeutic approach for various cancers, including HCC^10^. Immune checkpoint inhibitors (ICIs), which require an understanding of immunosuppressive roles of the immune system in the tumor microenvironment (TME), have greatly improved the clinical outcomes in multiple cancer types^11,12^. The TME, which is composed of non-cancerous stromal cells and noncellular components, plays a key role in tumor development and progression^13^. Several studies have explored the therapeutic potential and possible molecular mechanisms of costimulatory molecules, which have been shown to modulate tumor immunity^14,15^.

Costimulatory molecules and signals, which are composed of the B7-CD28 family and tumor necrosis factor (TNF) families, form a new and well-orchestrated regulation system of the tumor immune microenvironment, which is involved in various aspects of cancer biology and plays an essential role in immunotherapeutic strategies for various cancers, including HCC^16–18^. Most of the studies have focused on the potential therapeutic applications of costimulatory molecules in various cancers^17,19^, with only a handful of studies exploring their biological functions in HCC immunology. Therefore, we aimed at developing a potent and specific prognostic signature that is based on costimulatory molecules and signals with the main purpose of informing treatment decisions and improving the clinical outcomes for HCC patients.

Long noncoding RNAs (lncRNAs), which are located in the nucleus or cytoplasm, are non-coding RNAs with a length of about 200 nucleotides^20^. LncRNAs are associated with several stages of gene regulation, including chromatin modification, mRNA biogenesis, and protein signaling^21^. Moreover, lncRNAs have been shown to be prognostic markers for cancer and attractive targets for therapeutic interventions in the fight against various cancers^22^. For example, HULC, a lncRNA, promotes liver cancer cell tumorigenesis in vitro and in vivo by restraining PTEN via the ubiquitin–proteasome system under the mediation of autophagy-P62^23^. The expressions of W42 have been shown to be upregulated in HCC tissues and are associated with HCC cell proliferation and poor survival outcomes^24^. Overexpressed H19, which has been significantly correlated with poor prognostic outcomes for HCC patients, promotes HCC cell invasiveness by triggering and activating the miR-193b/MAPK1 axis^25^. Costimulatory molecules and signals are important regulatory pathways for tumors and other human diseases that are closely associated with lncRNAs^26^. Therefore, it is important to identify key lncRNAs that are closely associated with costimulatory molecules and prognosis of HCC patients, with the goal of improving the prognostic outcomes for HCC patients and providing potential therapeutic targets.

We systematically analyzed gene expressions in HCC using data obtained from The Cancer Genome Atlas (TCGA) and screened out costimulatory molecule‐related lncRNAs with prognostic values. Next, we constructed and validated a prognostic signature with five costimulatory molecule‐related lncRNAs from the TCGA cohort. Further analysis was conducted on AC099850.3, the costimulatory molecule‐related lncRNA that we found to have significant effects in HCC.

## Results

### Acquisition of costimulatory molecule-related lncRNAs

**Figure 1:**
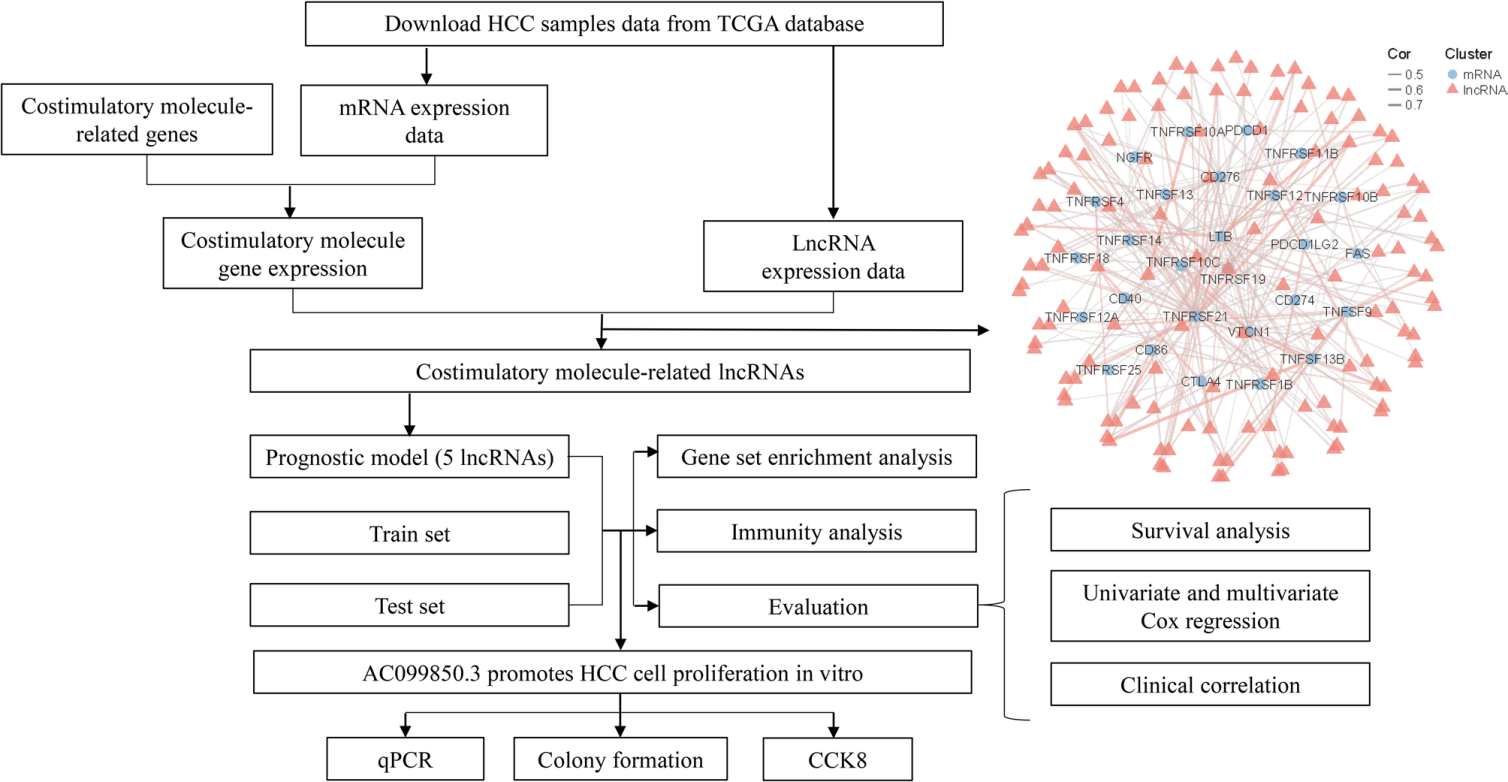
Flow chart.

Figure 1 shows the flow chart for the study. In total, 377 HCC samples and 50 adjacent non-tumor samples from the TCGA database were used in the analysis. Moreover, 59 costimulatory molecules were obtained from literature (Table 1). Finally, based on the screening criteria of |Correlation Coefficient|> 0.4 and *p* < 0.001, a total of 132 costimulatory molecule-related lncRNAs were identified from the TCGA-HCC data through construction of costimulatory molecule-related mRNA and lncRNA co‐expression network (Fig. 1).

**Table 1:** Costimulatory molecules.

Id	Aliases	Family
CD27	TNFRSF7	TNFRSF
CD274	PD-L1	B7
CD276	B7-H3	B7
CD28	Tp44	CD28
CD40	TNFRSF5	TNFRSF
CD40LG	TNFSF5	TNFSF
CD70	TNFSF7	TNFSF
CD80	B7-1	B7
CD86	B7-2	B7
CTLA4	CD152	CD28
EDA	EDA-A1	TNFSF
EDA2R	TNFRSF27	TNFRSF
EDAR	EDA-A1R	TNFRSF
FAS	TNFRSF6	TNFRSF
FASLG	TNFSF6	TNFSF
HHLA2	B7-H5	B7
ICOS	CD278	CD28
ICOSLG	B7-H2	B7
LTA	TNFSF1	TNFSF
LTB	TNFSF3	TNFSF
LTBR	TNFRSF3	TNFRSF
NGFR	TNFRSF16	TNFRSF
PDCD1	PD-1	CD28
PDCD1LG2	PD-L2	B7
RELT	TNFRSF19L	TNFRSF
TMIGD2	CD28H	CD28
TNF	TNFSF2	TNFSF
TNFRSF10A	TRAILR1	TNFRSF
TNFRSF10B	TRAILR2	TNFRSF
TNFRSF10C	TRAILR3	TNFRSF
TNFRSF10D	TRAILR4	TNFRSF
TNFRSF11A	RANK	TNFRSF
TNFRSF11B	OPG	TNFRSF
TNFRSF12A	FN14	TNFRSF
TNFRSF13B	TACI	TNFRSF
TNFRSF13C	BAFFR	TNFRSF
TNFRSF14	LIGHTR	TNFRSF
TNFRSF17	BCMA	TNFRSF
TNFRSF18	GITR	TNFRSF
TNFRSF19	TROY	TNFRSF
TNFRSF1A	TNFR1	TNFRSF
TNFRSF1B	TNFR2	TNFRSF
TNFRSF21	DR6	TNFRSF
TNFRSF25	DR3	TNFRSF
TNFRSF4	OX40	TNFRSF
TNFRSF8	CD30	TNFRSF
TNFRSF9	4-1BB	TNFRSF
TNFSF10	TRAIL	TNFSF
TNFSF11	RANKL	TNFSF
TNFSF12	TWEAK	TNFSF
TNFSF13	APRIL	TNFSF
TNFRSF6B	DCR3	TNFRSF
TNFSF14	LIGHT	TNFSF
TNFSF13B	BAFF	TNFSF
TNFSF15	TL1A	TNFSF
TNFSF18	GITRL	TNFSF
TNFSF4	OX-40L	TNFSF
TNFSF8	CD30L	TNFSF
TNFSF9	4-1BB-L	TNFSF
VTCN1	B7-H4	B7

### Identification of costimulatory molecule-related lncRNAs with significant prognostic values in HCC

**Figure 2:**
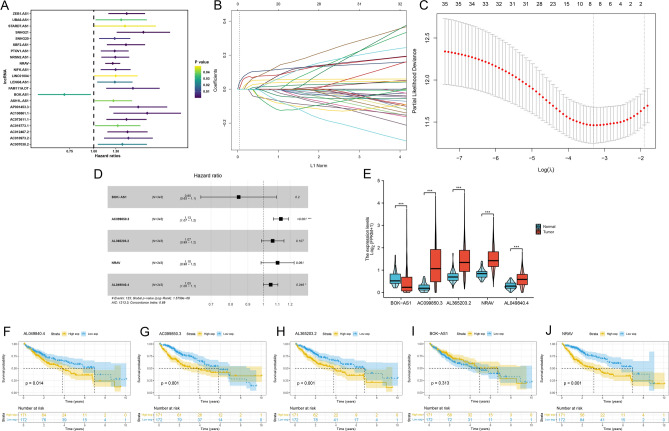
Identification of costimulatory molecule-related lncRNAs with prognostic values. **(A)** Results of univariate Cox regression analysis of prognostic costimulatory molecule-related lncRNAs in HCC patients. **(B, C)** LASSO regression analysis of costimulatory molecule-related lncRNAs selected from univariate Cox regression analysis. **(D)** Results of multivariate Cox regression analysis of prognostic costimulatory molecule-related lncRNAs in HCC patients. **(E)** Expressions of five prognostic costimulatory molecule-related lncRNAs in HCC and normal tissues. **(F)** Differential expression analyses of AC099850.3 in HCC and normal tissues from TCGA combined GTEx datasets. **(G)** Differential expression analysis of AC099850.3 in 50 normal-tumor paired samples. **(H)** Kaplan–Meier survival curves of five prognostic costimulatory molecule-related lncRNAs in the HCC cohort.

After excluding patients with a survival time of less than 30 days and with incomplete clinical data, 343 HCC patient samples were randomized into the training (*n* = 172) and testing (*n* = 171) cohorts (Table 2). Next, data was merged with costimulatory molecule-related lncRNAs to obtain clinical information and expressions of costimulatory molecule-related lncRNAs in HCC patients. Based on univariate Cox regression proportional hazards analyses, 36 costimulatory molecule-related lncRNAs that were associated with prognosis were selected (Table S1), including two low risk lncRNAs (hazard ration (HR) < 1) and 34 high risk lncRNAs (hazard ration (HR) > 1). The 22 lncRNAs with the lowest *p* values are shown in Fig. 2A. Furthermore, after 1000 iterations using LASSO Cox regression and multivariate Cox proportional hazards regression analyses, 31 OS-related lncRNAs were identified (Fig. 2B,C). Finally, we constructed a risk prognostic signature consisting of five OS-related costimulatory molecule-related lncRNAs, including BOK-AS1, AC099850.3, AL365203.2, NRAV, and AL049840.4 (Fig. 2D). Coefficients for each costimulatory molecule-related lncRNA were obtained from the model (Table S2). Next, expressions of prognostic lncRNAs between HCC and adjacent non-tumor samples were analyzed (Fig. 2E), which revealed significant variations in their expressions. In addition, expressions of AC099850.3 were upregulated in most of the HCC tissues in GSE67260 and GSE84005 series (Table S3). Differential expression analysis was performed on 160 normal samples and 371 tumor samples from TCGA combined GTEx datasets. The expressions of all five signature lncRNAs between tumor and normal samples were markedly different, among which AC099850.3 was the most significantly differentially expressed lncRNA (Figure S1A-E). Meanwhile, analysis of the 50 normal-tumor paired samples revealed significantly higher AC099850.3 levels in tumor samples (Figure S1F). Finally, Kaplan–Meier (K–M) survival analysis was performed to compare the OS time between the high- and low-expression groups for each prognostic lncRNA (Fig. 2H).

**Table 2:** Patients’ clinical features of training set and testing set.

Covariate		Total	Training set	Testing set
		*N* = 343	*N* = 172	*N* = 171
Age (years), no (%)	≤ 65	216 (63.0)	55 (32.0)	99 (57.9)
	> 65	127 (37.0)	117 (68.0)	72 (42.1)
Gender (years), no (%)	Female	110 (32.1)	53 (30.8)	57 (33.3)
	Male	233 (67.9)	119 (69.2)	114 (66.7)
Vital status, no (%)	Alive	220 (64.1)	110 (64.0)	110 (64.3)
	Dead	123 (35.9)	62 (36.0)	61 (35.7)
Grade, no (%)	G1	53 (15.5)	22 (12.8)	31 (18.1)
	G2	161 (46.9)	85 (49.4)	76 (44.4)
	G3	112 (32.7)	55 (32.0)	57 (33.3)
	G4	12 (3.5)	8 (4.7)	4 (2.3)
	Unknow	5 (1.5)	2 (1.2)	3 (1.8)
Stage, no (%)	Stage I-II	238 (69.4)	115 (66.9)	123 (71.9)
	Stage III-IV	83 (24.2)	43 (25.0)	40 (23.4)
	Unknow	22 (6.4)	14 (8.1)	8 (4.7)
T state, no (%)	T1	168 (49)	84 (48.8)	84 (49.1)
	T2	84 (24.5)	40 (23.3)	44 (25.7)
	T3	75 (21.9)	39 (22.7)	36 (21.1)
	T4	13 (3.8)	8 (4.7)	5 (2.9)
	TX	3 (0.9)	1 (0.6)	2 (1.2)
N stage, no (%)	N0	239 (69.7)	119 (69.2)	120 (70.1)
	N1	3 (0.9)	1 (0.6)	2 (1.2)
	NX	101 (29.4)	52 (30.2)	49 (28.7)
M stage, no (%)	M0	245 (71.4)	122 (70.9)	123 (71.9)
	M1	3 (0.9)	2 (1.2)	1 (0.6)
	MX	95 (27.7)	48 (27.9)	47 (27.5)

### Evaluation and verification of the prognostic signature containing the five OS-related lncRNAs

**Figure 3:**
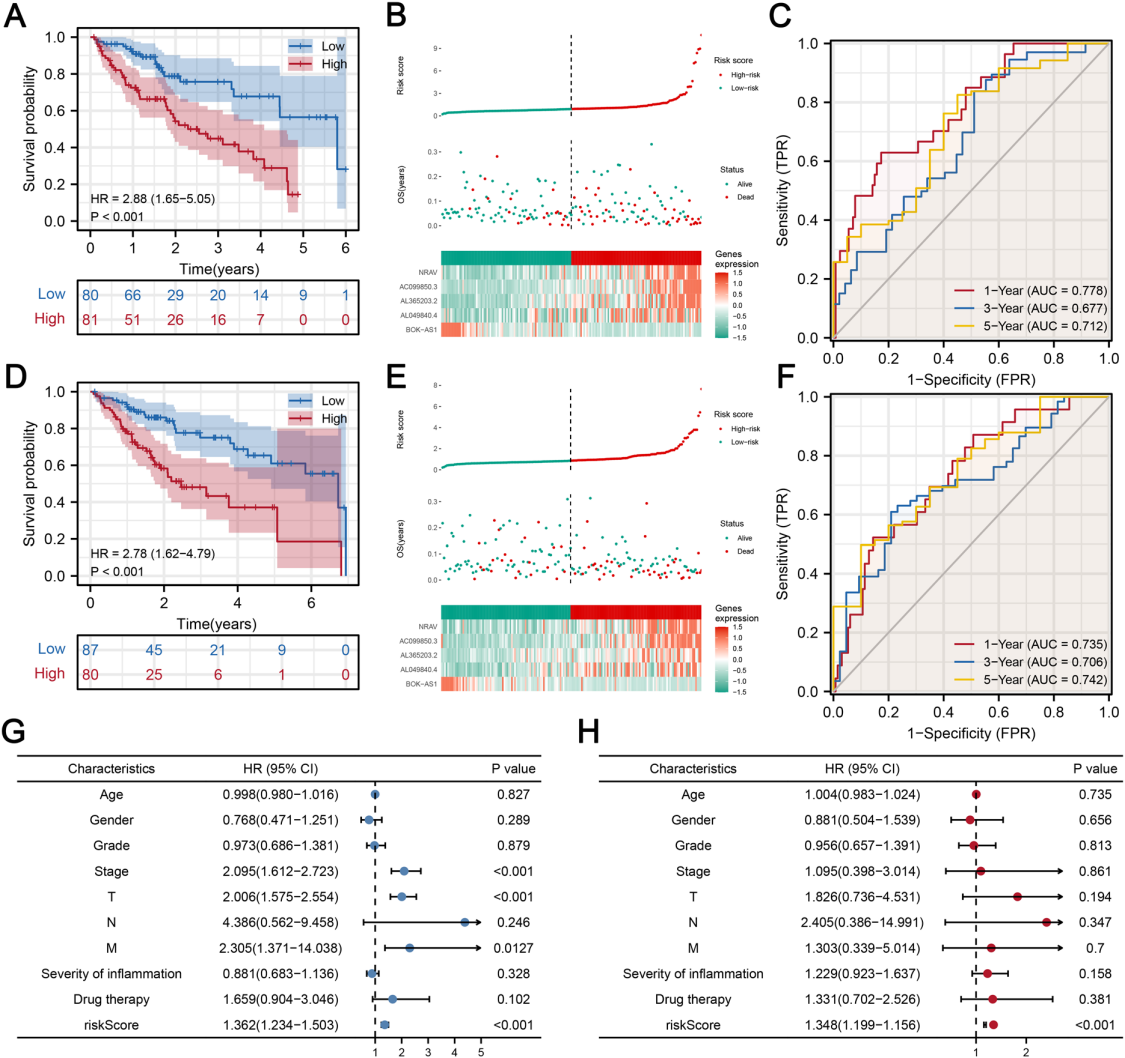
Evaluation and verification of the prognostic costimulatory molecule-related lncRNA signature. Kaplan–Meier survival curves for high- and low-risk patients in training **(A)** and testing **(D)** cohorts. Distribution and survival status of HCC patients with different risk scores. A heatmap showing the expression levels of five prognostic costimulatory molecule-related lncRNAs in the high- and low-risk groups in training **(B)** and testing **(E)** cohorts. Time-dependent ROC curves of the prognostic signature in training **(C)** and testing **(F)** cohorts. **(G, H)** Univariate and multivariate Cox regression analyses for the risk score as an independent prognostic factor.

Based on the risk-score formula and the calculated median risk score, patients in training and testing cohorts were classified into high‐ and low‐risk score groups. Kaplan–Meier survival analysis revealed that in both training and testing cohorts, the OS time for the high-risk score group was significantly shorter than that of the low-risk score group (Fig. 3A,D; HR = 2.88 (1.65–5.05), *p* < 0.001 in training cohort and HR = 2.78 (1.62–4.79), *p* < 0.001 in testing cohort), indicating that the risk-score can predict the prognostic outcomes for HCC patients (all *p* < 0.001). Distributions of risk scores and survival statuses for each patient were visualized by the risk curve and scatterplot, which showed that patient mortality was associated with risk scores (Fig. 3B,E). The heatmap for the five OS-related lncRNA expressions in HCC samples showed that NVAR, AC099850.3, AL3652.3.2, and AL049840.4 are potential risk factors, and all were highly expressed in the high‐risk group. Besides, BOK-AS1 was found to be a potential protective factor that was upregulated in the low‐risk groups of the training and testing cohorts (Fig. 3B,E). The time-dependent ROC curve was calculated to assess the predictive sensitivity and specificity of the risk score on the prognostic outcomes for HCC patients. The AUC values for 1‐, 3‐, and 5-years were 0.778, 0.677, and 0.712 in the training cohort and 0.735, 0.706, and 0.742 in the testing cohort, respectively (The best cut-offs for 1‐, 3‐, and 5-years were 1.192, 0.834, and 0.806 in the training cohort and 1.408, 0.946, and 0.946 in the testing cohort, the sensitivities for 1‐, 3‐, and 5-years were 0.629, 0.838, and 0.825 in the training cohort and 0.522, 0.609, and 0.497 in the testing cohort, while the specificities for 1‐, 3‐, and 5-years were 0.415, 0.488, and 0.550 in the training cohort and 0.856, 0.791, and 0.900 in the testing cohort) (Fig. 3C,F). Thus, the five costimulatory molecule-related lncRNAs were reliable for constructing the prognostic risk model for HCC. Then, univariate and multivariate Cox regression analyses were performed to determine whether the five costimulatory molecule-related lncRNAs could be used as independent prognostic biomarkers for HCC patients. Univariate Cox regression analysis revealed that stage (*p* < 0.001, 95% CI 1.612–2.723), T (*p* < 0.001, 95%CI 1.575–2.554), and risk-score (*p* < 0.001, 95%CI 1.234–1.503) were associated with prognosis. However, only the risk-score (*p* < 0.001, 95%CI 1.179–1.476), rather than inflammation severity in adjacent hepatic tissues and whether patients received drug treatment, was associated with prognostic outcomes in multivariate Cox regression analysis. These results imply that the risk model of the five costimulatory molecule-related lncRNAs is an independent prognostic factor for HCC patients (Fig. 3G-H). Finally, given that OS time for patients is highly dependent on disease stages, Kaplan–Meier survival analysis was performed for patients in different stages. High risk patients exhibited worse prognostic outcomes in different disease stages (Figure S2). Collectively, the prognostic signature of the five costimulatory molecule-related lncRNAs is a significant independent prognostic factor for HCC patients. Then, gene set enrichment analysis (GSEA) was performed between the high‐ and low‐risk score groups to identify the potential signaling pathway. Figure S3 shows the different hallmark functions, Kyoto Encyclopedia of Genes and Genomes (KEGG) pathways and gene ontology (GO) pathways between the high- and low-risk groups. The high‐risk score group correlated with cancer, while the low‐risk score group correlated with enhanced oxidation.

### Immunity analysis of HCC

**Figure 4:**
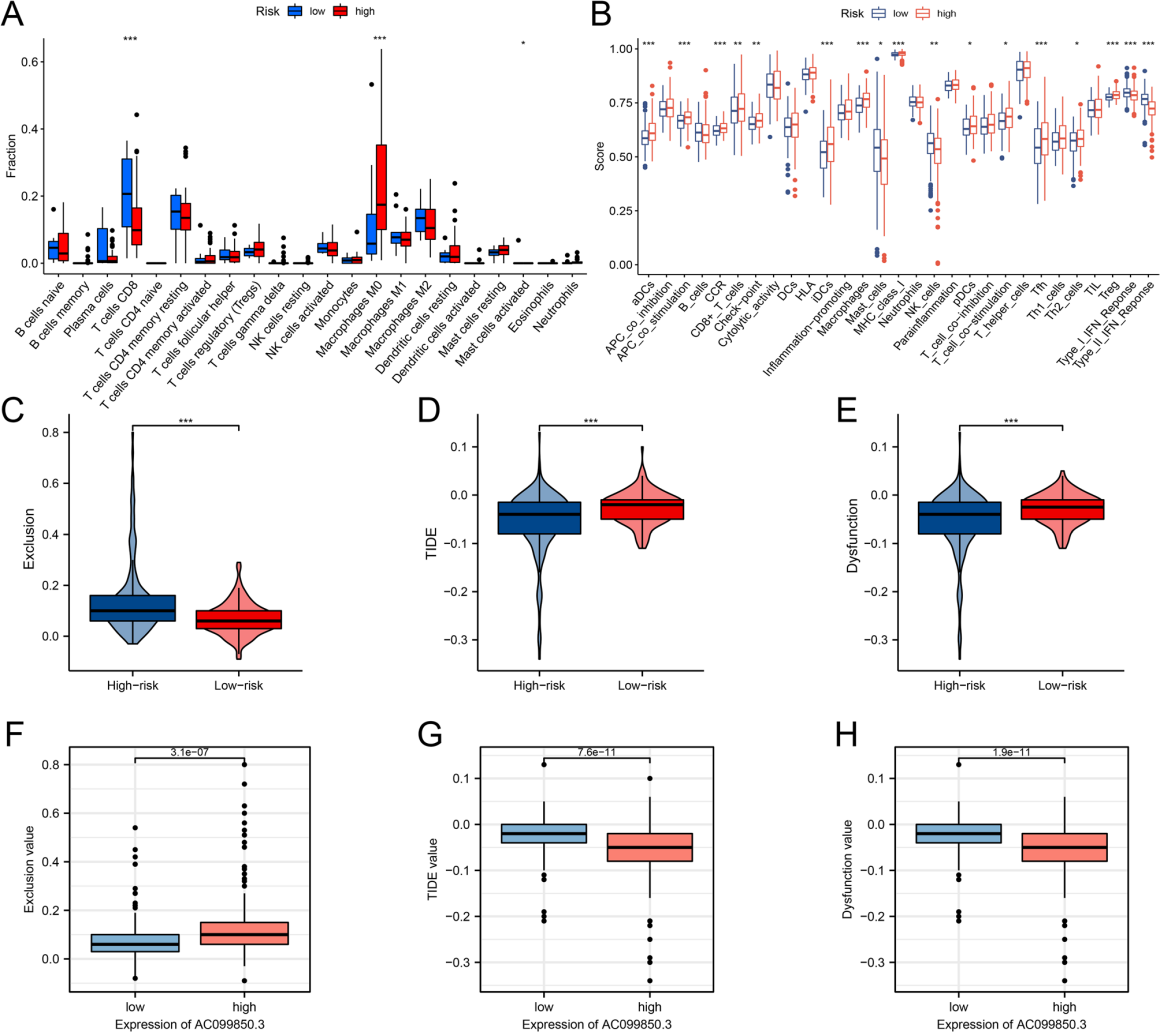
Patients with high- and low-risk scores had different immune status. Comparisons of ssGSEA scores for 22 immune celltypes **(A)** and 29 immune-related pathways **(B)** between low- and high-risk groups. **(C)** High immune exclusion score values in the high-risk group. Comparisons of TIDE **(D)** and immune dysfunction **(E)** scores for chemotherapeutics and targeted therapy between the two risk groups. Patients with high-risk scores were more suitable for immunotherapy. **(F–H)** TIDE analysis showing that patients with low AC099850.3 expressions had a low exclusion value as well as higher TIDE and dysfunction values.

To elucidate on the effects of high and low expression levels on immune cell infiltrations and functions between the two risk stratifications, based on the costimulatory molecule-related lncRNA signature, the TIMER 2.0 database was used to quantify immune infiltrations. Figure 4A shows that tumor-infiltrating CD8 + T cell proportions were significantly low in high-risk patients while expressions of the M0 macrophage were high in high-risk patients, compared to low-risk patients. Correlation analyses between immune cell subpopulations and related functions revealed that T cell functions (Tfh, Treg, type I IFN responses, and type II IFN responses), and aDCs, iDCs, and CCR were significantly low in high-risk groups, relative to low-risk group (Fig. 4B). These findings suggest that infiltrations of these immune cell types and their related immune functions might play a major role on the prognostic outcomes of HCC patients. Given the significance of immune checkpoint blockade-based therapy for HCC, the Tumor Immune Dysfunction and Exclusion (TIDE, http://tide.dfci.harvard.edu) online tool was used to evaluate the association between risk stratifications and the effects of immune checkpoint inhibition therapy (Fig. 4C-E). TIDE analysis revealed that patients with suppressed AC099850.3 levels had higher TIDE values (Fig. 4F-H).

### Clinical correlation analysis and immunity features of AC099850.3

**Figure 5:**
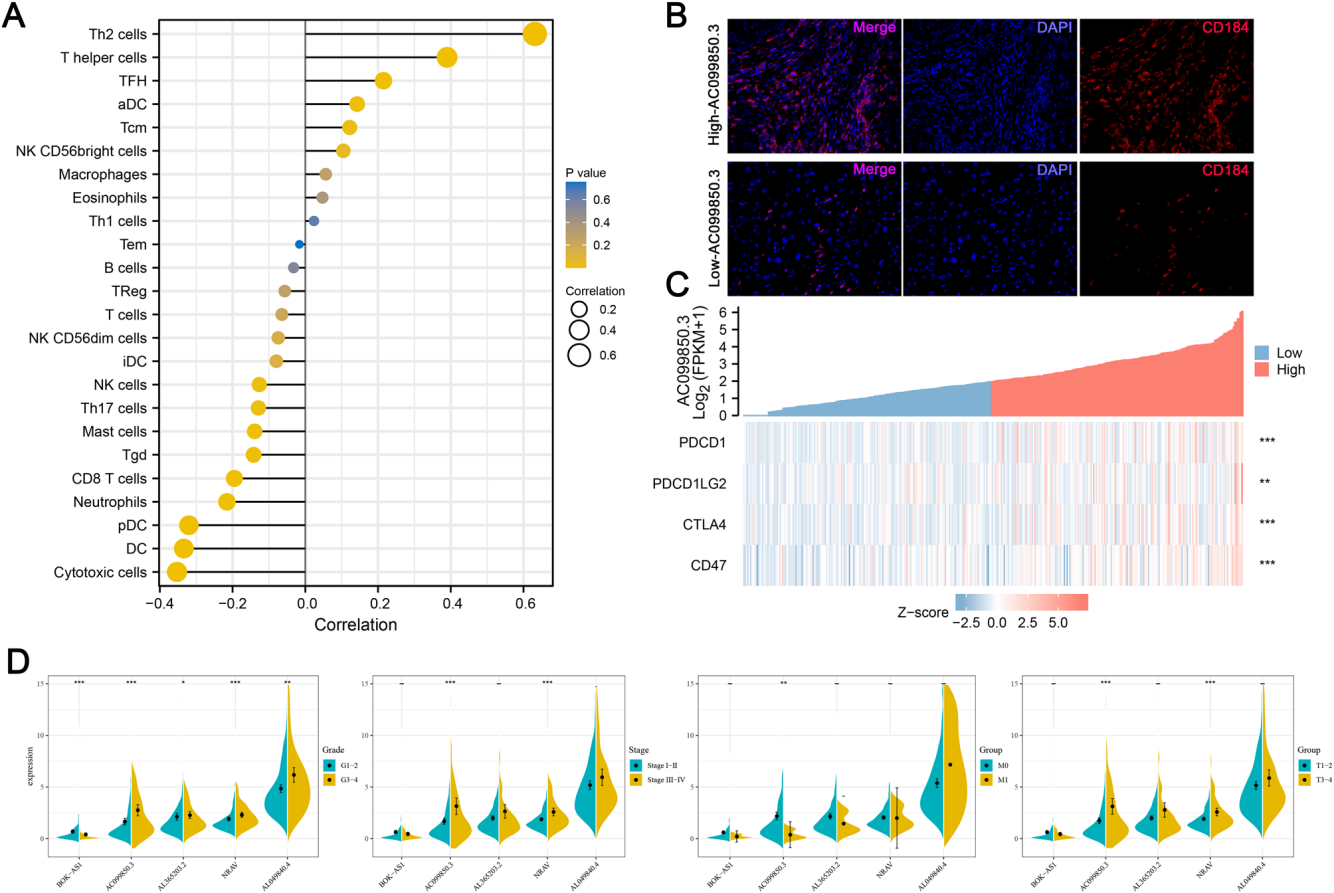
Clinical value of AC099850.3 and its association with immune features for HCC patients. **(A)** Lollipop-diagram showing that AC099850.3 was strongly correlated with immune cells, including Th2 cells and T helper cells. **(B)** Representative immunofluorescence images indicated different CD184 levels in high and low AC099850.3 expressing tissues. **(C)** Heatmap showing positive association between AC099850.3 expressions and several immune checkpoints. **(D)** Violin plot demonstrating that AC099850.3 was strongly associated with HCC progression, including Grade, Stage, T stage and M stage, compared to other prognostic costimulatory molecule-related lncRNAs. *FPKM* Fragments Per Kilobase of exon model per Million mapped fragments.

Multivariate Cox analysis revealed that AC099850.3 was the most significant lncRNA for OS, therefore, it was selected for further research. Figure 5A shows the results from ssGSEA analysis, which revealed highly significant correlations between AC099850.3 levels and Th2 cells and T helper cells. Immunofluorescence showed that AC099850.3 expressions had significantly positive correlations with Th2 cells and a strong-to-moderate correlation with T helper cells (Fig. 5B). GSEA analysis was performed between high and low AC099850.3 groups to identify the potential biological signaling pathway difference, in which we found that the E2F targets, G2M checkpoint, mitotic spindle and PI3K-AKT-mTOR pathways were significantly activated in high-AC099850.3 patients, suggesting the proliferation-promoting effect of AC099850.3 might be dependent on these pathways (Figure S4). Given that immune checkpoint modules play important roles in the tumor environment, correlation analysis between AC099850.3 and four immune checkpoints were performed. Patients with elevated AC099850.3 levels exhibited higher levels of these immune checkpoints (Fig. 5C). To investigate whether the five costimulatory molecule-related lncRNAs are involved in HCC development, we assessed the significance between expressions of the five costimulatory molecule-related lncRNAs with clinicopathological parameters. There was a significant association between AC099850.3 expressions and clinicopathological factors for HCC patients, including grade, AJCC stage, and TNM stage (Fig. 5D). Then, correlation analysis was performed between Th2 cell and T helper cell levels with AC099850.3 expressions and the other lncRNAs in HCC development. Patients in the high risk score and worse pathologic stage (Grade 3–4) groups exhibited higher levels of Th2 cells (Figure S5A-D). Moreover, T helper cell levels were higher in high risk score patients, compared to low risk score patients (Figure S5E-H). Furthermore, among the five signature lncRNAs, AC099850.3 expressions were strongly correlated with Th2 cells (Figure S5I), while correlations between T helper cells with AC099850.3 and other lncRNAs were not strong (Figure S5J). In addition, analysis of expressions of the five signature lncRNAs was performed in training and testing cohorts to evaluate the underlying interactions (Figure S6A-B). Further analyses were conducted on correlations between the other lncRNAs and immune cell types (Figure S6C-F).

### AC099850.3 promoted HCC cell proliferation in vitro

#### AC099850.3 expressions were upregulated in HCC tissues and cell lines

**Figure 6:**
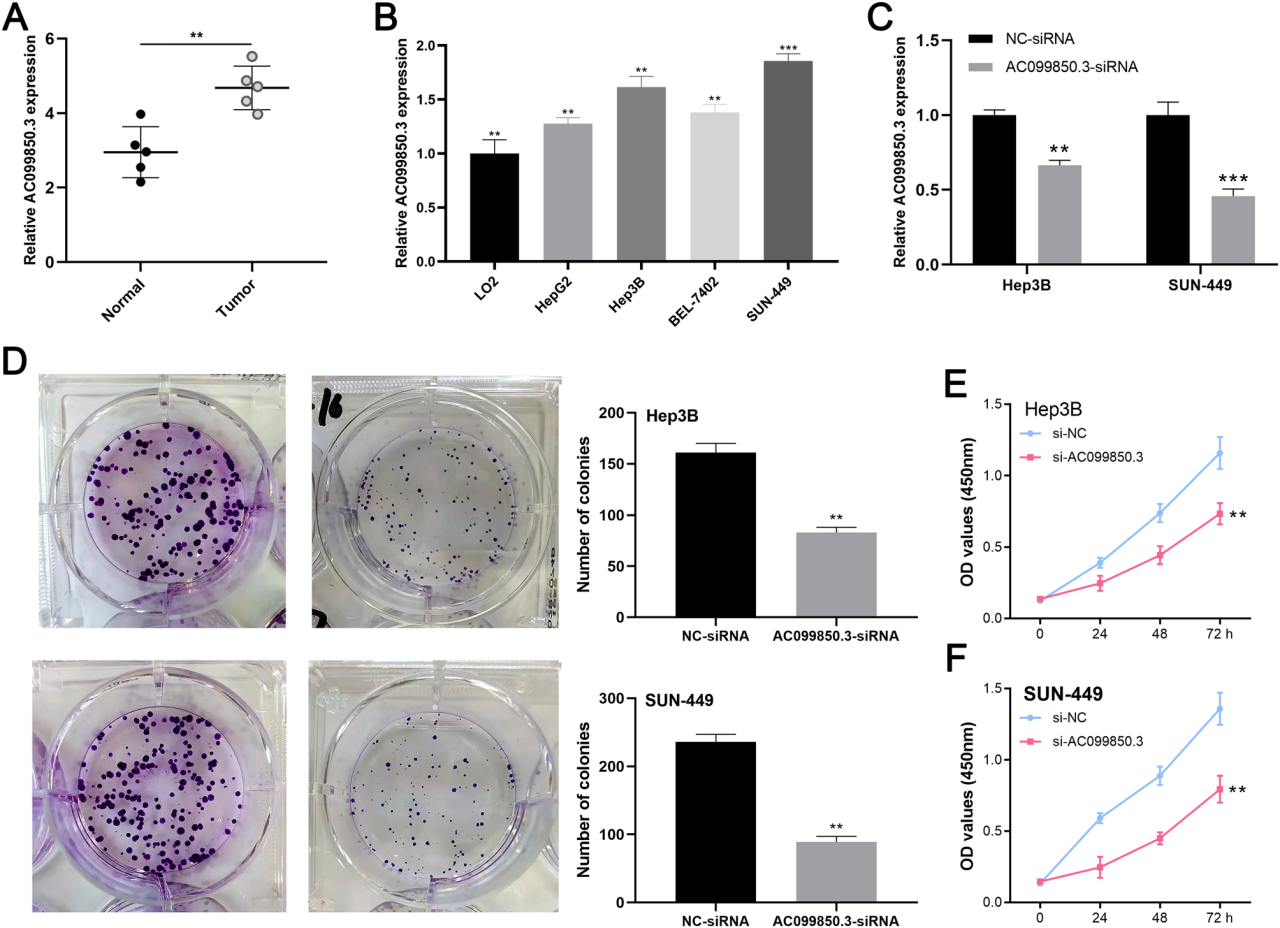
AC099850.3 promoted HCC cell proliferation in vitro. **(A)** AC099850.3 mRNA expressions in tumors and non-malignant tissues from HCC patients. **(B)** AC099850.3 mRNA expressions in HCC cell lines and normal hepatic cells. **(C)** mRNA expressions of AC099850.3 in cell lines with AC099850 knockdown. **(D)** Colony formation abilities of Hep3B and SUN-449 cells after AC099850.3 knockdown. Results of CCK8 assay for Hep3B **(E)** and SUN-449 cells **(F)** after AC010973.2 knockdown.

Based on the five paired HCC tissues, qRT-PCR analyses showed that AC099850.3 levels were significantly upregulated in tumoral tissues (*p* < 0.01, Fig. 6A), which was also validated at the cell level (Fig. 6B). Given that AC099850.3 levels were highest, Hep3B and SUN-449 cells were transfected with target sequences against AC099850.3 for further research. AC099850.3 knockdown was verified by determination of their mRNA expressions (Fig. 6C).

#### AC010973.2 promoted HCC cell proliferations

The colony formation assays revealed that clonogenic survival was significantly decreased in the si-AC099850.3 group, relative to the control group in both Hep3B and SUN-449 cell lines (Fig. 6D), suggesting that AC099850.3 knockdown weakened the ability of tumor cells to proliferate. Similar results were observed in the CCK8 assays, indicating that AC099850.3 knockdown significantly inhibited the proliferative capacity of HCC cell lines (Fig. 6E,F). Therefore, AC099850.3 expressions promote HCC cell growth and survival, presumably by favoring cancer cell survival in the tissue environment.

## Discussion

Globally, hepatocellular carcinoma (HCC), which accounts for approximately 90% of all liver cancers, is one of the most common malignancies with a high probability for metastasis and recurrence^2^. Given that most HCC patients are diagnosed at the intermediate and advanced stages when surgical resection is not an option, immunotherapy and systemic therapies remain the optimal treatment approaches for HCC patients. However, these therapeutic options are associated with various side effects, a heavy financial burden and worse prognostic outcomes^27^. Therefore, there is an urgent need to determine sensitive and reliable prognostic biomarkers for identifying patients with poor prognostic outcomes and those who can benefit from early adjuvant treatment, instead of salvage treatment. In recent years, an increasing number of studies have focused on molecular characteristics for early diagnosis and prognosis. Some functional lncRNAs can be used to elucidate on the initial process of malignant cancer progression, which may inform the development of more efficient measures to improve prognostic outcomes^28^. Some costimulatory molecule-related signatures are significantly associated with clinical features and can stratify patients into two subgroups with different prognoses, which can guide treatment^29^. However, only a handful of studies have evaluated the potential therapeutic and prognostic roles of costimulatory molecule-related lncRNAs in HCC.

In this study, the Cox regression model identified a novel costimulatory molecule-related five-lncRNAs signature in a TCGA cohort, which was sensitive and specific. Multivariate Cox regression analysis revealed that among the five lncRNAs signature, only the risk-score was an appropriate independent predictive factor for HCC patients and was significantly correlated with different clinicopathological parameters. Our prognostic signature was also associated with the tumor immune microenvironment and immunotherapeutic responses, which provides valuable information for predicting prognostic outcomes for HCC patients and guiding immunotherapy. In addition, the colony formation assays showed that AC099850.3 knockdown significantly inhibited the proliferative capacities of HCC cell lines.

Costimulatory molecules play an important role in regulation of tumor immunity. For instance, discovery of immune checkpoints in the B7-CD28 family, one of the two main costimulatory molecule families, has opened new possibilities for inducing durable tumor regressions using monoclonal antibodies (mAb)^30^. Kanodia et al*.*^31^ reported that overexpressions of the costimulatory molecule, TNFSF14, enhanced the expansion of tumor antigen-specific T-cells, thereby inhibiting the proliferation of human papillomavirus 16-induced tumors by altering the tumor microenvironment. Various lncRNAs are associated with liver cancer development and progression, and have the potential to be used in diagnosis, prognosis, and therapy. For instance, the long noncoding RNA, lncTCF7, was found to be highly expressed in HCC tumors, and could promote self-renewal and proliferation of liver cancer stem cells (CSCs) by activating Wnt signaling^32^. In a previous study, expressions of lnc-DILC were suppressed in HCC patients and were correlated with IL-6 and CD24 levels, suggesting that lnc-DILC is a potential prognostic biomarker and therapeutic target against liver CSCs^33^. Wang et al*.*^34^ performed a comprehensive bioinformatics analysis involving liver cancer patients and identified a four-lncRNAs prognostic signature that can specifically predict the prognostic outcomes for liver cancer patients and improve clinical outcomes.

We identified 132 costimulatory molecule-related lncRNAs from TCGA-HCC by constructing a related mRNA and lncRNA co‐expression network. Lasso and Cox proportional hazards regression analyses revealed five costimulatory molecule-related lncRNAs (BOK-AS1, AC099850.3, AL365203.2, NRAV, and AL049840.4) with prognostic values. Among them, AC099850.3, AL365203.2, NRAV, and AL049840.4 were risk factors that were upregulated in the high‐risk score group, whereas BOK-AS1 was a protective factor that was downregulated in the high‐risk score group. AC099850.3 was significantly upregulated in HCC patients, and could promote HCC cell migration as well as proliferation, suggesting that elevated AC099850.3 levels are markers for poor prognostic outcomes for HCC patients^35^. The lncRNAs, AL365203.2 and NRAV, are associated with poor prognostic outcomes, and can regulate the infiltrations of numerous immune cell types in HCC as well as its progression^36^. Notably, the functions of BOK-AS1 and AL049840.4 in HCC have not been identified. These costimulatory molecular-related lncRNAs are novel, which warrants further studies to explore their roles in HCC.

In this study, cancer-related pathways, including epithelial mesenchymal transitions, which is involved in multiple signal transduction pathways and is closely associated with tumor cell invasion and metastasis, were significantly enriched in the high-risk group^37^. Moreover, the cell cycle can protect tumor cells from different stresses and promote tumor progression, whereas the B cell receptor signaling pathway can activate the expressions of genes involved in B cell proliferation, differentiation, and other tumor processes^38^. This suggests that the five costimulatory molecular-related lncRNAs identified in this study are associated with HCC occurrence and development. To explore the associations between our signature and the tumor immune microenvironment, immune cell infiltration types and their related immune functions were compared between the two risk stratifications. We found that tumor-infiltrating CD8 + T cells, T cell functions (Tfh, Treg, type I IFN response, and type II IFN response), aDCs, iDCs, and CCR were significantly low in the high- risk group, compared to the low-risk group, whereas expressions of M0 macrophages and NK cells were higher in high-risk patients, compared to the low-risk group. In a previous study, percentages of exhausted CD8 + T cells were found to be significantly increased in liver cancer samples and late stage patients exhibited higher exhaustion levels than other patients, which confirmed its association with poor prognostic outcomes for liver cancer patients^39^. Moreover, a high abundance of macrophages in colorectal liver metastasis patients are associated with worse prognostic outcomes^40^. Infiltrations of a large number of Treg cells is often associated with poor prognostic outcomes, which is a challenge for immunotherapeutic efficacies^41^. The TIDE score, a newly-developed computational method that is used to model tumor immune evasion, is a more accurate biomarker than TMB or PD-L1 expression^42^. In this study, high-risk patients exhibited high exclusion scores, whereas dysfunction and TIDE scores were low, implying that high-risk patients may be suitable for immunotherapy. As the most critical model lncRNA, AC099850.3 was selected for further research. Immune dysfunction could lead to the proliferation ability difference in cancer cells. If the proliferation caused by AC099850.3 was related to immune dysfunction, a higher dysfunction value should be observed in high AC099850.3 groups. However, our results seem not to support this. In our study, a lower immune dysfunction value was found in high AC099850.3 patients, indicating that the proliferation-promoting effect of AC099850.3 might be dependent on other manners rather that immune dysfunction. Therefore, ssGSEA combined with correlation analysis was adopted to investigate the relationship between AC099850.3 expression and immune infiltration levels in HCC, which demonstrate that the expressions of AC099850.3 were significantly positively correlated with Th2 cells, which play vital roles in HCC metastasis^43^. Th2 cell is a type of T helper cell that can produce IL-4, resulting in activation of several cancer-related pathways^44^. We think it might partly explain the cancer-promoting effect of AC099850.3 to some extent. In conclusion, overexpressed AC099850.3 has a key role in immune infiltrations during HCC progression. With regards to clinical relevance between AC099850.3 levels and clinicopathological parameters, AC099850.3 was highly expressed in the latter stages of HCC, including grade 3–4, stage III–IV, and T3–4, implying that AC099850.3 is a biomarker for poor prognostic outcomes. QRT-PCR was performed to validate whether AC099850.3 promotes HCC development. It was found that AC099850.3 levels were significantly upregulated in tumor tissues. Besides, colony formation and CCK8 assays revealed that AC099850.3 knockdown significantly inhibited HCC cell proliferations.

In conclusion, this study established a risk signature of HCC based on five costimulatory molecule-related lncRNAs for the first time, which could be used to stratify patients for accurate prediction of the prognostic outcomes of HCC patients. Our study was an exploratory analysis. Here, we identified some novel lncRNAs related to costimulatory molecules that have not been previously reported in HCC and these lncRNAs had considerable potential in the prognosis and treatment of HCC patients and had important reference value for later researchers. In addition, we found that AC099850.3 could significantly promote proliferation of HCC cells. However, this study had some limitations. Firstly, due to the lack of lncRNA expression profile and incomplete clinical information, it is hard for us to validate it in other external cohorts except TCGA. Therefore, our study should be further validated in other prospective cohorts in the future. Secondly, the crosstalk between AC099850.3 and immune cells in TME should be further explored, and functional experiments should be conducted to elucidate the potential molecular mechanisms of the five lncRNAs associated with costimulatory molecules, especially AC099850.3.

## Materials and methods

### Data preparation and sources

All liver cancer cases in this study were exclusively hepatocellular carcinoma (HCC). Data for HCC patients, including RNA sequencing normalized as FPKM and clinical information, were downloaded from The Cancer Genome Atlas (TCGA) database (https://portal.gdc.cancer.gov/). The data were from 377 HCC tissues and 50 normal tissues. Data from the GTEx database (https://www.gtexportal.org/) was used to validate the results. Costimulatory molecules associated with HCC (Supplementary Table S2) were identified from literature^17^.

### Screening of costimulatory molecule-related lncRNAs in HCC

First, 59 costimulatory molecules were identified from literature. Then, Pearson’s correlation coefficient analysis was performed to determine correlations between lncRNA levels and the corresponding costimulatory molecules using “limma” R package^45^. The lncRNAs were regarded as being related to costimulatory molecules based on the following criteria: |Correlation Coefficient|> 0.4 and *p* < 0.001.

### Construction of the costimulatory molecule-related lncRNAs prognostic signature

After excluding patients with missing clinical information and survival time less than 30 days to eliminate non-cancer related deaths, 343 HCC samples were randomized into the training (*n* = 172) and testing (*n* = 171) cohorts. The clinicopathological parameters for HCC patients, including age, gender, stage, TNM stage, grade, and cancer status of HCC are shown in Supplementary Table S1. The training cohort was used to construct the prognostic signature, followed by validation in the testing cohort. Based on the criteria of *p* < 0.05, Univariate Cox regression analysis was performed to determine the costimulatory molecule-related lncRNAs that were associated with overall survival outcomes (OS, defined as the time from registration to death from any cause) for HCC patients in the training group. Then, lasso regression analysis was conducted using the “glmnet” R package with the optimal value of penalty parameter (λ) determined according to the tenfold cross-validations that were used to select significant features^46^. Finally, multivariate Cox regression analysis was performed to construct a prognostic model and lncRNAs with independent prognostic predictive values were enrolled for construction of the risk-score model in accordance with the formula:

$$riskscore={\sum }_{i=1}^{k}coef\left(lncRNAi\right)*\mathrm{exp}(lncRNAi)$$

whereby, exp (lncRNAi) indicates the expressions of lncRNA while coef (lncRNAi) is the correlation coefficient of the lncRNA in the risk-score model.

### Evaluation and verification of the prognostic signature

The above formula was used to calculate the risk score for each HCC patient, followed by stratifying the patients into high‐ and low‐risk score groups based on their prognostic risk scores.

Given that the OS for HCC are relatively shorter, patients whose OS ranged between 1 and 6 years were selected for Kaplan‐Meier survival analysis to compare the OS time between the high- and low-risk groups. To estimate the models’ predictive accuracy, the time-dependent receiver operating characteristic (ROC) and the area under ROC curve (AUC) for 1‐, 3‐, and 5-years were plotted. Furthermore, distribution curves, scatter dot plot, and heatmap were used to visualize risk-score distributions, number of censored patients, and prognosis‐related lncRNAs in the two groups. Univariate and multivariate Cox regression analyses between risk scores and different clinical factors (age, gender, grade, stage, TNM stage, severity of inflammation in adjacent hepatic tissues, and whether patients received drug treatment) were performed using the “survival” R package to assess whether the risk-score is an independent indicator for HCC prognosis.

### Gene set enrichment analysis

To investigate the pathways and biological processes in which the prognostic lncRNAs are involved in, gene set enrichment analysis (GSEA) was performed to the two risk stratifications of the molecular-related lncRNA prognosis signature using “h.all.v7.4.symbols.gmt”, “c5.all.v7.2.symbols.gmt” and “c2.cp.kegg.v7.4.symbols.gmt” packages in the GSEA software.

### Evaluation of immune features based on the prognostic signature

Immune cell data was obtained from the Tumor Immune Estimation Resource (TIMER) database, and used to determine the relationship between immune cell infiltration types as well as their related immune functions and risk-scores^47^. Next, densities of the 22 immune cell types in the tumor were evaluated after which activities of the 29 immune-related functions between the high‐risk score group and low‐risk score group were determined. To predict immunotherapeutic responses for each sample, transcriptome profiles from the TCGA datasets were analyzed using the Tumor Immune Dysfunction and Exclusion (TIDE, http://tide.dfci.harvard.edu) online tool.

### Analysis of the correlation between prognostic lncRNAs and clinicopathological parameters

Among the five lncRNAs, AC099850.3 was primarily expressed in HCC tissues. After multivariate Cox analysis, it was found to be the most significant lncRNA, with the lowest *p* value for OS. Therefore, to assess the significance of AC099850.3 on correlations with clinicopathological parameters, violin plots were generated to visualize differential expressions and clinicopathological factors. Then, a heatmap and a lollipop-diagram were generated to show correlations between immune functions and AC099850.3. Immunofluorescence was performed to validate the impact of AC099850.3 on Th2 cells. The CD184 primary antibody was purchased from proteintech (Proteintech Group, Inc). The secondary antibody was goat anti-mouse IgG heavy and light chain.

### Quantitative real-time PCR (qRT-PCR) assay for expressions of AC099850.3 in HCC cell lines

Human HCC cell lines (HepG2, Hep3B, BEL-7402, and SUM-449) and the normal hepatic cell line (LO2) were purchased from the Cell Bank of the Chinese Academy of Sciences (Shanghai, China) and used for qRT-PCR analysis. Briefly, total cellular RNA was extracted using an RNA extraction kit, as instructed by the manufacturer. The RNA was reverse-transcribed to cDNA using a reverse transcription kit (TaqMan), in accordance with the manufacturer’s instructions. Then, the qPCR assay was conducted using SYBR Green methods. The primer sequences used in this study were: AC099850.3, forward: 5′-CTGGAGTGGCAGTGTTGCAATC-3′; AC099850.3, reverse: 5′-GGTGACGCACACCTGTAGTCC-3′; GAPDH, forward: 5′-GAAGGTGAAGGTCGGAGTC-3′; GAPDH, reverse: 5′-GAAGATGGTGAT GGGATTTC-3′.

### Colony formation assay

The Hep3B and SUN-449 cells were transfected with lncRNA-targeted siRNAs for the clonogenic assay. Next, 500 cells were plated in six-well plates and incubated for a minimum of 14 days. Cell colonies were counted after crystal violet staining.

### Cell proliferation assay

The CCK-8 (Dojindo, Shanghai, China) assay was performed to evaluate cell proliferation abilities. Briefly, cells were plated in 96-well plates (2000 cells/well) and cultured at 37 °C for 24 h, 48 h, and 72 h. After adding 10% CCK8 reagent into the corresponding wells, absorbance at 450 nm was measured to evaluate cell proliferation abilities.

## Conclusions

In conclusion, we developed and validated a novel costimulatory molecule-related survival model consisting of five lncRNAs (BOK-AS1, AC099850.3, AL365203.2, NRAV, and AL049840.4). Moreover, it was established that AC099850.3 can promote HCC proliferation. The above five costimulatory molecule-related lncRNAs are potential therapeutic and prognostic targets for HCC.

## Supplementary Information

Supplementary Information 1.

Supplementary Information 2.

Supplementary Information 3.

Supplementary Information 4.

Supplementary Information 5.

Supplementary Information 6.

Supplementary Information 7.

Supplementary Information 8.

Supplementary Information 9.

Supplementary Information 10.
