# Supplementary material for: Identification of a novel prognostic signature for HCC and analysis of costimulatory molecule-related lncRNA AC099850.3
Source: Sci Rep. 2022 Jun 15;12:9954. doi: 10.1038/s41598-022-13792-z (PMC9200812; doi:10.1038/s41598-022-13792-z)
Supplement: Supplementary file 1 — Supplementary Information 1. [file 41598_2022_13792_MOESM1_ESM.docx]

**Supplementary figure legends**

**Figure S1: Differential expression analysis of the five signature lncRNAs between tumor and normal samples.**

**Notes: (A-E)** Expressions of the five signature lncRNAs between tumor and normal samples were markedly different, among which AC099850.3 was the most significantly differentially expressed lncRNA. **(F)** Differential expression analysis of the five signature lncRNAs between 50 normal-tumor paired samples.

**Figure S2: Kaplan-Meier survival analysis for patients in different stages.**

**Figure S3: Gene set enrichment analysis for the costimulatory molecule-related lncRNA signature.**

**Notes:** The top ten enriched gene sets for the high- and low-risk groups using gene sets of HALLMARK **(A,D)** KEGG **(B,E)** and GO **(C,F)**.

**Figure S4: GSEA analysis between high and low AC099850.3 groups.**

**Figure S5: Associations of AC099850.3 and lncRNAs expressions with Th2 cells and T helper cells.**

**Notes: (A-D)** Connection analysis of Th2 cell levels, riskscore and HCC development. **(E-H)** Connection analysis of T helper cell levels, riskscore and HCC development. **(I)** Expressions of AC099850.3 were most strongly correlated with Th2 cells among the five signature lncRNAs **(J)** Connection analysis of T helper cell levels and the five signature lncRNAs.

**Figure S6: Associations of the 5 lncRNAs and costimulatory molecules with immune cell types.**

**Notes: (A-B)** Connection analysis between expressions of the 5 signature lncRNAs in training and testing cohorts. **(C-F)** Correlations between other four lncRNAs and immune cell types.
