# Supplementary figures and images for: Identification of a novel prognostic signature for HCC and analysis of costimulatory molecule-related lncRNA AC099850.3
Source: Sci Rep. 2022 Jun 15;12:9954. doi: 10.1038/s41598-022-13792-z (PMC9200812; doi:10.1038/s41598-022-13792-z)

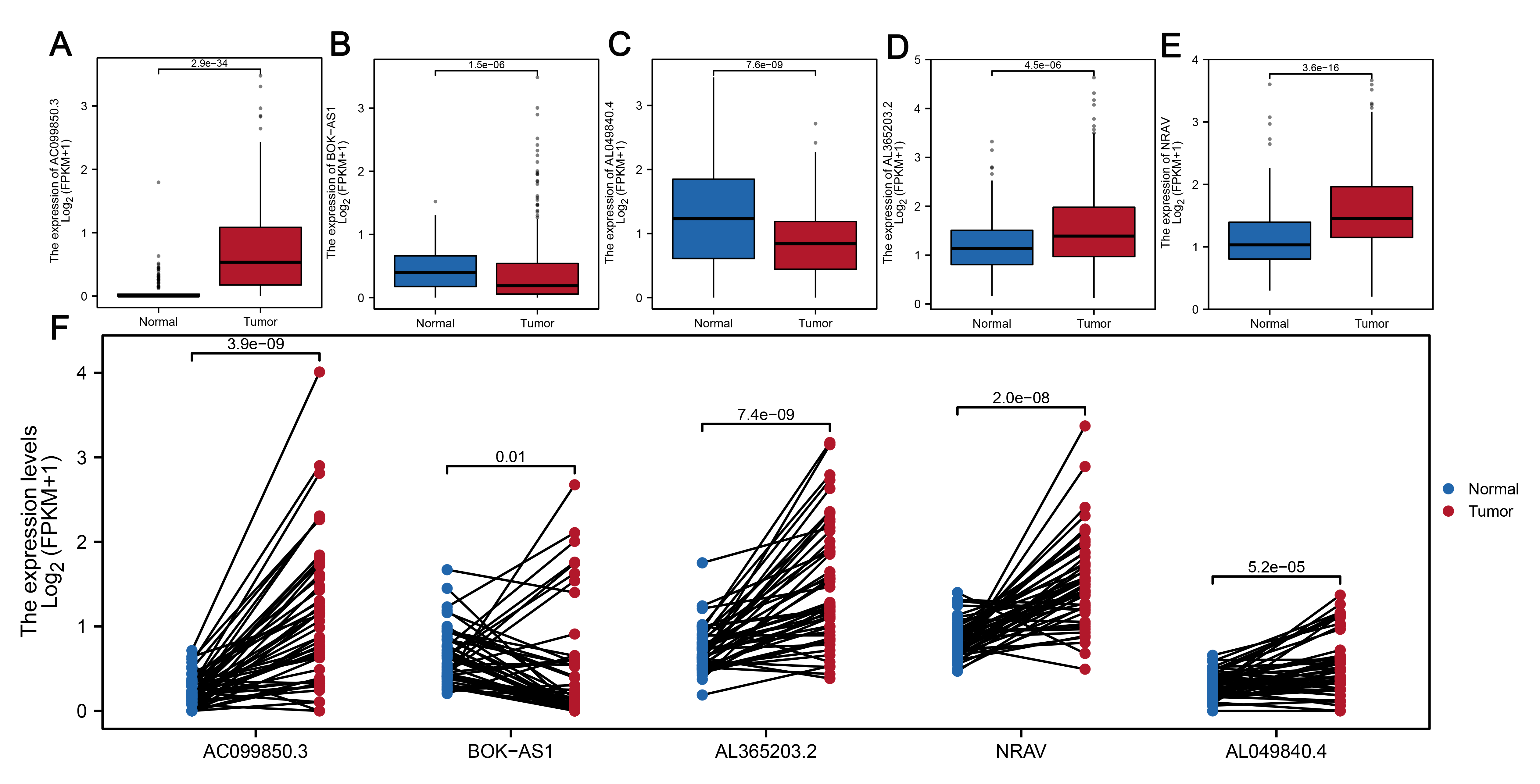

Supplement: Supplementary file 2 — Supplementary Information 2. [file 41598_2022_13792_MOESM2_ESM.tif]

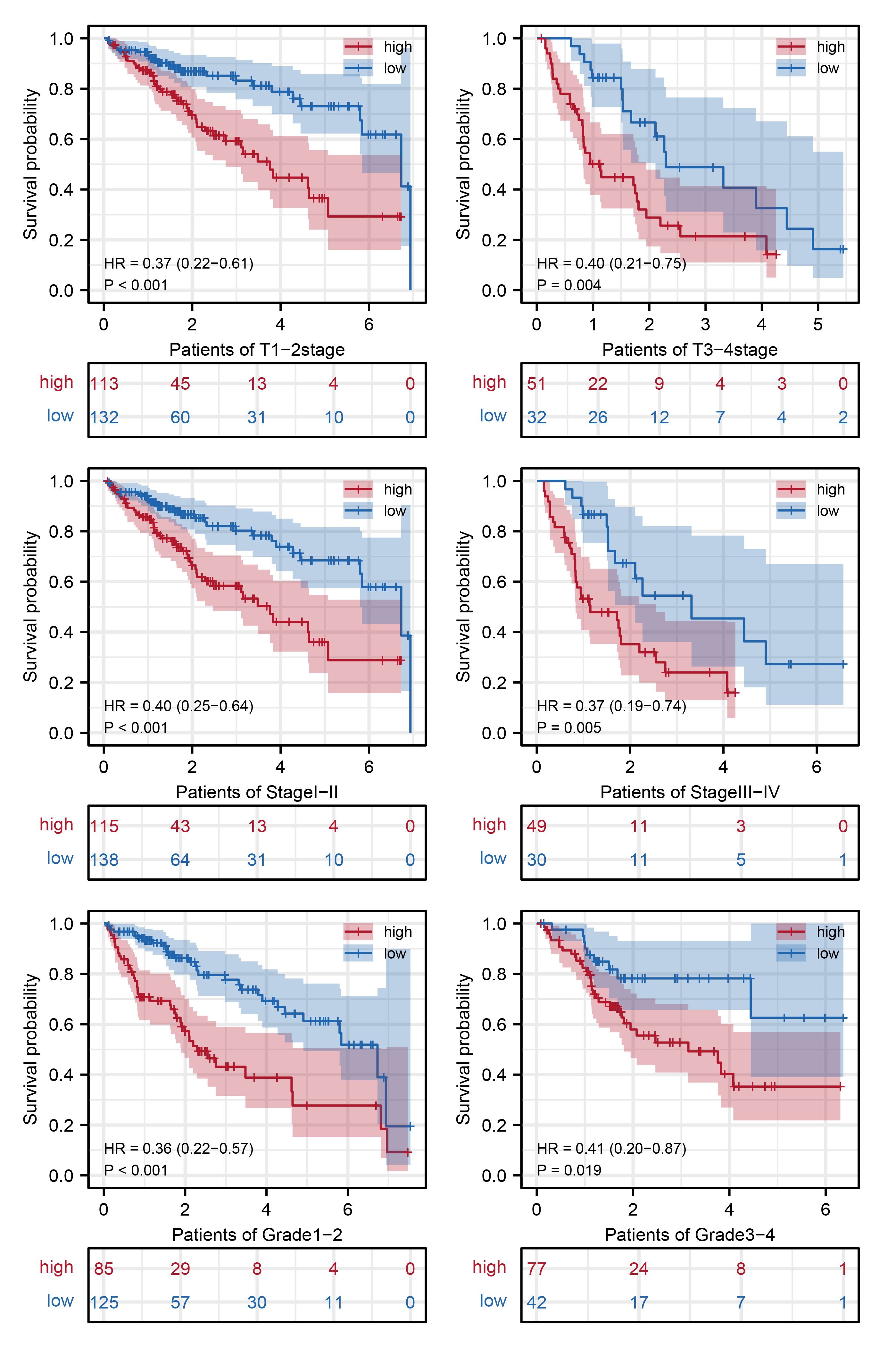

Supplement: Supplementary file 3 — Supplementary Information 3. [file 41598_2022_13792_MOESM3_ESM.tif]

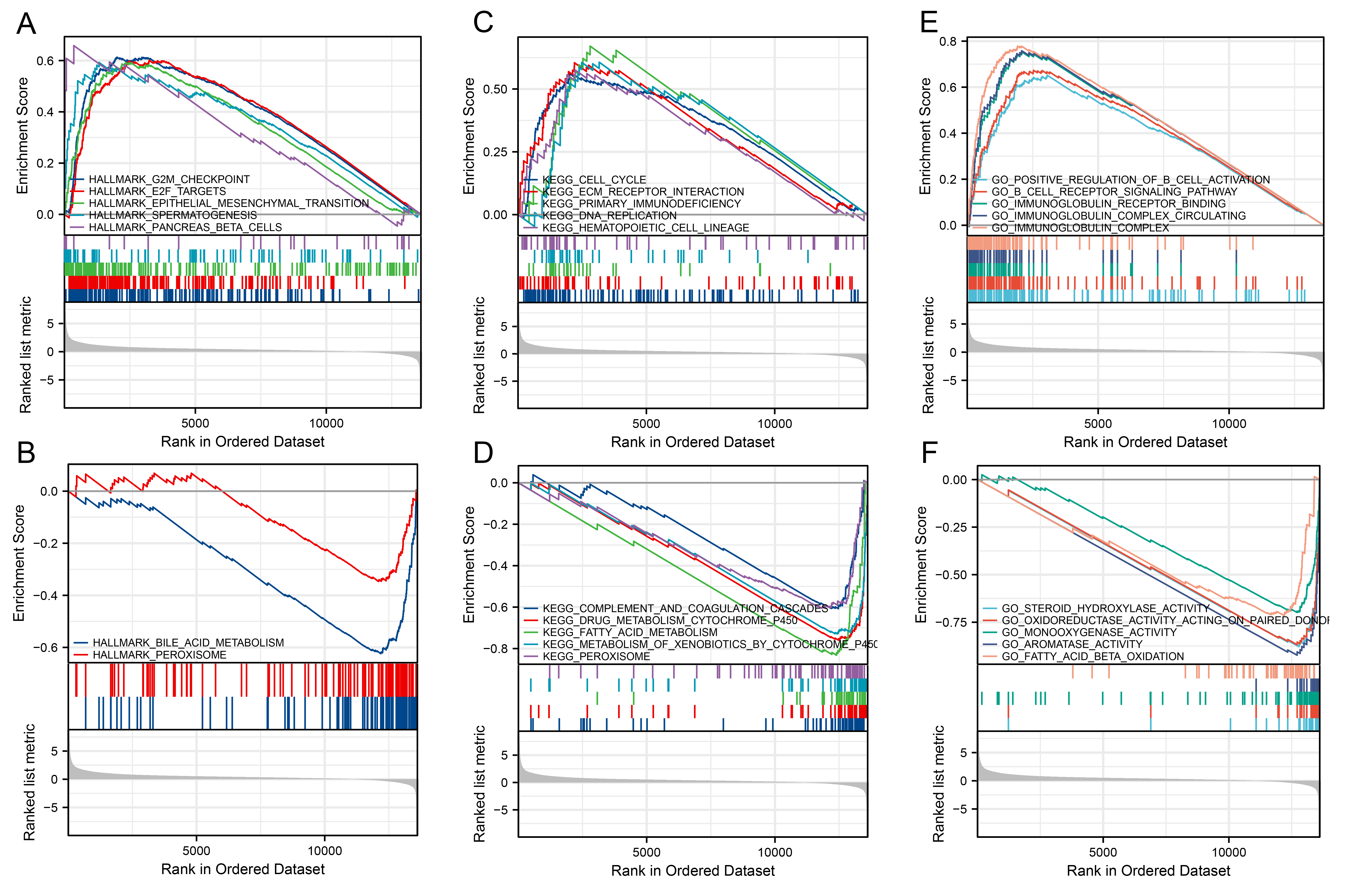

Supplement: Supplementary file 4 — Supplementary Information 4. [file 41598_2022_13792_MOESM4_ESM.tif]

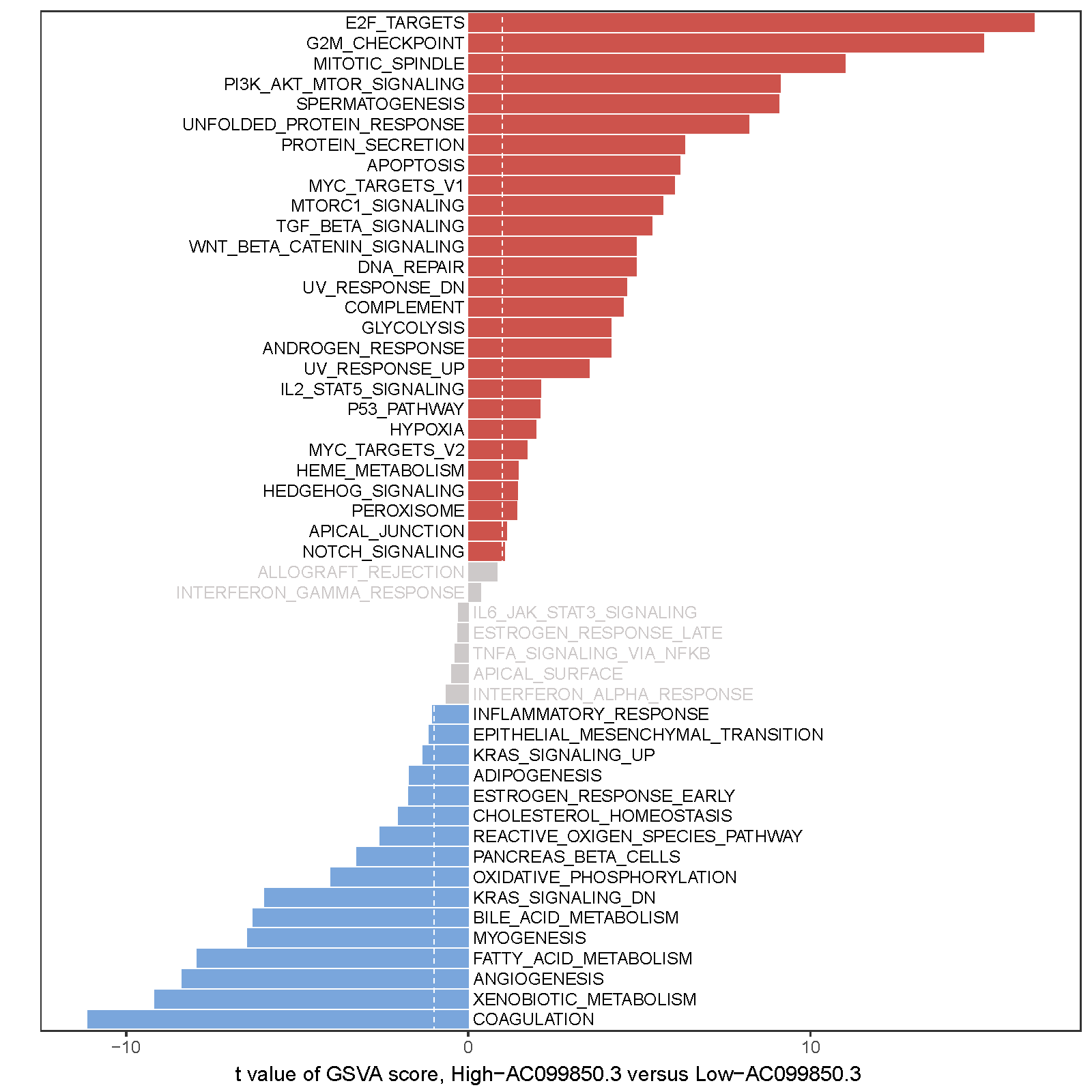

Supplement: Supplementary file 5 — Supplementary Information 5. [file 41598_2022_13792_MOESM5_ESM.tiff]

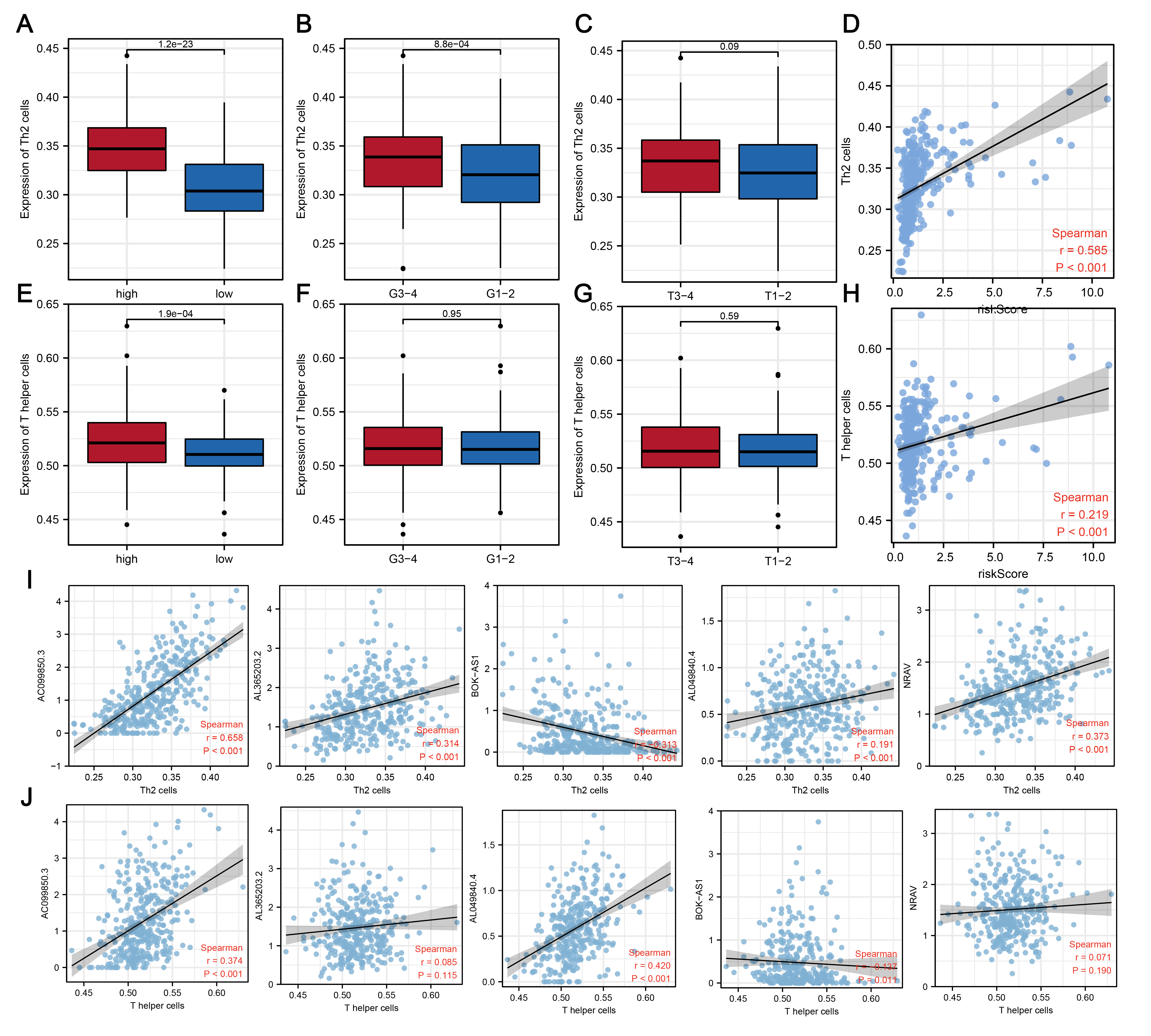

Supplement: Supplementary file 6 — Supplementary Information 6. [file 41598_2022_13792_MOESM6_ESM.tif]

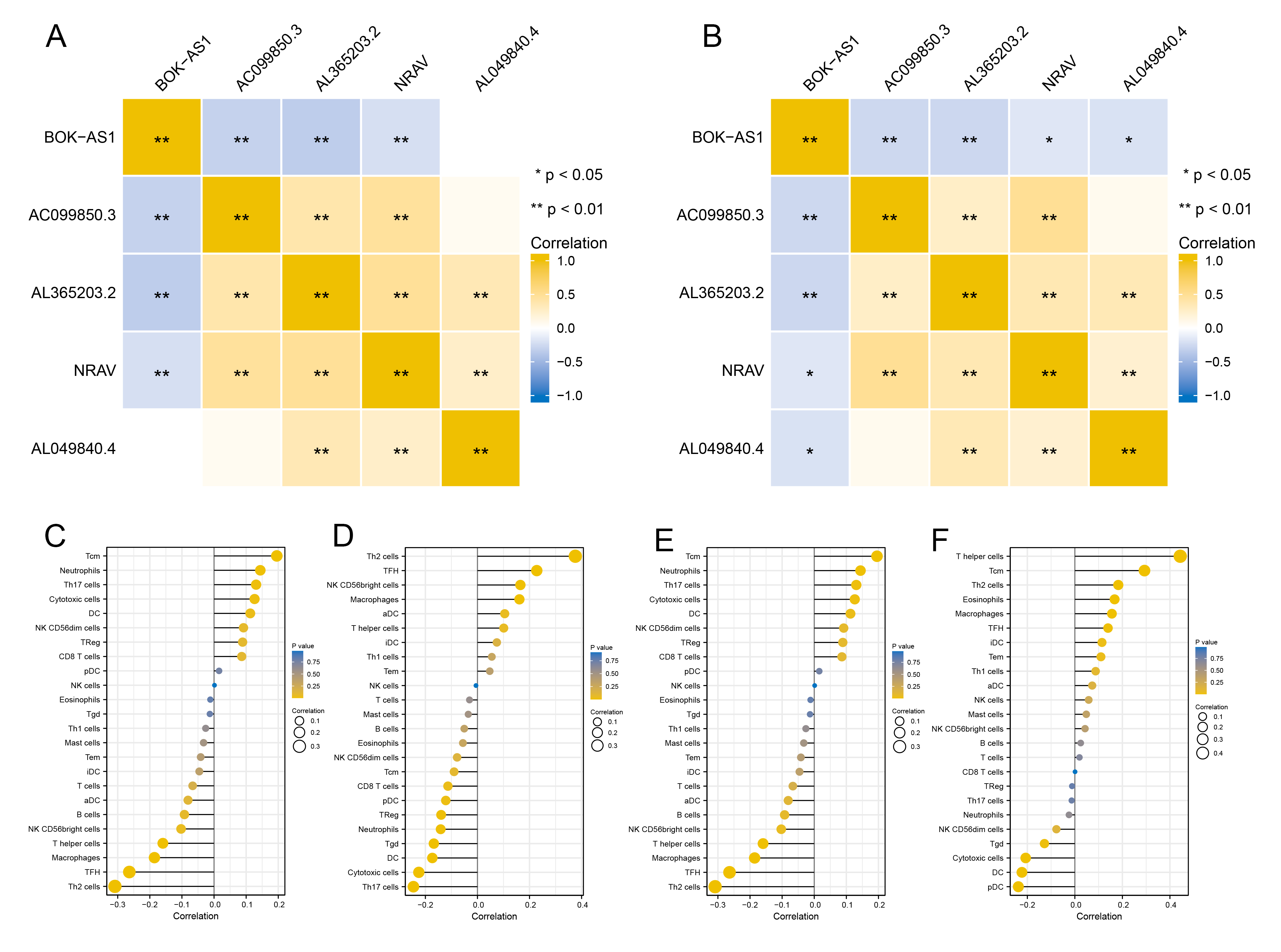

Supplement: Supplementary file 7 — Supplementary Information 7. [file 41598_2022_13792_MOESM7_ESM.tif]
